# Total Phenolic Content and Antibacterial Activity of Five Plants of Labiatae against Four Foodborne and Some Other Bacteria

**Published:** 2014

**Authors:** Arash Mahboubi, Mohammad Kamalinejad, Abdul Majid Ayatollahi, Mohammad Babaeian

**Affiliations:** a *Department of Pharmaceutics, School of Pharmacy, Shahid Beheshti University of Medical Sciences, Tehran, Iran. *; b *School of Pharmacy, Shahid Beheshti University of Medical Sciences, Tehran, Iran.*; c *Department of Pharmacognosy, School of Pharmacy, Shahid Beheshti University of Medical Sciences, Tehran, Iran.*

**Keywords:** Thymus, Ziziphora, Zataria, Antibacterial, Foodborne, Phenolic content, Folin-Ciocalteu

## Abstract

The aim of this study was to evaluate the antibacterial effects of *Thymus vulgaris*, *Thymus caramanicus*, *Zataria multiflora*, *Ziziphora clinopodioides* and *Ziziphora tenuior* against four foodborne and four other bacteria including *Staphylococcus aureus*, *Shigella dysenteriae*, *Salmonella typhimurium*, *Escherichia coli*, *Staphylococcus epidermidis*, *Bacillus subtilis*, MRSA and *Pseudomona aeruginosa* and measuring the amount of total phenolics of the plants.

The extracts were prepared by maceration method. Pre-evaluation of the antimicrobial effect was utilized by cup-plate technique and then Minimum Inhibitory Concentration was determined by agar dilution method according to NCCLS. The total phenolics as a possible cause of antibacterial effect, was measured by Folin-Ciocalteucolorimetry.

The results showed that *T. caramanicus *and *Z. multiflora *were the most effective ones with MIC values between 0.78-3.125 mg/mL against all of the Bactria and *Z. tenuior *and Z. clinopodioides had the minimum antimicrobial activity. Total phenolic contents of these five plants were different and followed the general pattern of the antimicrobial effect.

The antibacterial effects and the total phenolic content of *T. caramanicus* and *Z. multiflora* were remarkable and should be investigated more in future studies.

## Introduction

Food poisoning is usually assumed as an acute disease caused by contaminated food or beverages, with the bacteria as its major cause (1,2). Other factors which can cause such disease are parasites, toxic plants, fungi and *etc *(2,3). Annually about 76 million persons are affected worldwide and most of the cases occur in summer. Generally children and old people are at a higher risk (2). World Health Organization has estimated that in 2005, 1.8 million people have died due to diarrheal diseases and a considerable amount of these cases were after consumption of contaminated food and water (4).

*Rotavirus* and *Escherichia coli* are the two most common causes of diarrhea in developing countries (5). Another pathogen that usually spreads through contaminated food and water is *Shigella *(6). Other bacterial agents such as *Staphylococcus aureus*,* Bacillus cereus*,* Clostridium perfringens*,* Clostridium botulinum*,* Vibrio cholerae*,* Campylobacter jejuni,* and *Vibrio parahaemolyticus* are involved in the incidence of food poisoning (7,8). Foodborne disease is usually mild but can also be fatal (7). These diseases may have long-term and variable symptoms such as watery or bloody diarrhea, meningitis, chronic renal disorders, and respiratory, immunologic and cardiovascular complications (9,10). In Iran, the main pathogens causing diarrhea seems to have a different pattern in comparison to the many developed countries. In Iran *Escherichia coli* has been reported as the most common cause of diarrhea and Shigella is the most common cause of bacterial dysentery. Because pork is not consumed in Iran, Yersiniosis is not common (11). In 2006, 26 foodborne illness outbreaks have been reported in Iran, but some researchers said that the extent of the problem is certainly much greater; the most commonly found causative agents were Eschericia coli, non-typhi Salmonella*, *Salmonella typhi*, *Shigella*, *Staphylococcus aureus*, *Entamoeba histolytica, and *Rotavirus *(12).

Today, chemicals are used widely to prevent or delay food spoilage. There are evidences of carcinogenic and toxic effects of these preservatives. So food manufacturers and consumers should be careful applying such materials (13). Recently due to increased consumer awareness, growing interest to use natural preservatives such as essential oils and natural materials with antimicrobial effects has been occurred (14,15).

In this study five plants of Labiatae family including* Thymus vulgaris*, *Thymus caramanicus*, *Zataria multiflora*, *Ziziphora clinopodioides* and *Ziziphora tenuior* with fairly similar properties in botanical characteristics and applications (3,16-25) were investigated. There are some reports of similarities in their chemical composition too (16-24,26). These plants are commonly known and used to relieve many diseases such as gastrointestinal disorders. *In-vitro* antimicrobial effect of the essential oils and extracts of some of these five plants have been confirmed against some species of bacteria. Essential oil and extract of *T. vulgaris (*Avishan-e-Baghi) have shown inhibitory effect against *Bacillus cereus*, *Salmonella*, *Proteus* and Eschericia coli (27-29). Several studies showed antimicrobial effect of essential oil of* Z. clinopodioides* (Avishan-e-Barik) and its extract against pathogens such as *Bacillus subtilis*, *Salmonella*, *Klebsiella*, *Streptococcus faecalis*, *Enterobacteraerogenes* and Eschericia coli (30-33). A few studies confirmed antimicrobial effect of the essential oil of *T. caramanicus* (Avishan-e-Kermani) against some bacteria like *Staphylococcus aureus*, *Bacillus subtilis*, Eschericia coli and antimicrobial effect of its extract against Helicobacter pylori has been investigated (34,35). Antimicrobial effects of essential oil of *Z. tenuior* (Kakouti) against several bacteria such as *Staphylococcus aureus*, *Bacillus subtilis*, *Klebsiella*, Eschericia coli and *Proteus* have been studied (26). Studies show that essential oil and extract of *Z. multiflora* (Avishan-e-Shirazi) have antimicrobial activity against some bacteria such as *Salmonella*, *Staphylococcus aureus*, *Bacillus cereus*, *Proteus*, *Shigella flexneri*, *Enterococcus faecalis* and *Klebsiella *(17,18,36,37). Crude *T. vulgaris* is used commonly in gastrointestinal disorders and as an antimicrobial agent (19). In Iranian folk medicine leaves of *T. caramanicus* is used in treatment of Rheumatism, skin disorders and as an antibacterial agent (19,35). In Iran *Z. clinopodioides*is known as drug, spice and antimicrobial agent among people (20,21). Decoction of *Z. multiflora*is used to relieve gastric complications and common cold popularly (20). *Z. tenuior* is used to relieve common cold symptoms and gastrointestinal complications traditionally and like Zataria and some types of thymus as condiments for yoghurt and doogh (3,20,22-24,38).

According to pattern of foodborne pathogens in Iran and lack of available studies on the antimicrobial effects of these plants against foodborne bacteria with considering the common use of these five plants as food additives in Iran (20,39), the antimicrobial effects of hydroalcoholic extracts of these five medicinal plants against some foodborne pathogens including *Staphylococcus aureus*, *Shigella dysenteriae*, *Salmonella typhimurium* and *Escherichia coli* and four more bacteria including *Staphylococcus epidermidis*, *Bacillus subtilis*, Methicillin-resistant *Staphylococcus aureus* (MRSA) and *Pseudomona aeruginosa* were investigated. In order to explain the antibacterial effects of the extracts, their phenolic content as possible root of effects were determine by Folin-Ciocalteu colorimetric method.

## Experimental

*Collection of medicinal plants and spices*

*T. vulgaris, T. caramanicus, Z. multiflora*,* Z. clinopodioides* and *Z. tenuior* were collected from botanical garden of research center of Agriculture in Tehran, Karkas Mountain in Natanz, Jahrom, Shahre-babak mountains and Khodabandeh respectively and were identified and confirmed in medicinal plants laboratory of Department of Pharmacognosy, school of pharmacy, Shahid Beheshti university of Medical Sciences, Tehran, Iran. Aerial parts of the plants stored in a dry and dark place for about 72 h to get dried.

*Extraction procedure*

The extracts obtained by maceration; 200 g of powdered aerial parts of each plant were immersed in 1000 mL of ethanol (Merck, Germany) and water in equal proportions. Samples were mixed for 48 h on a shaker (Heidolph, Germany) at room temperature. The extraction of the plants samples were repeated for 2 more times. The solvent of the extract was evaporated by rotary evaporator (Heidolph, Germany). Dry extracts were stored in sterile and light protected containers and at 4 °C.

*Bacterial strains and culture media*

Lyophilized bacteria; *S. aureus*(ATCC: 6538), *S. typhimurium*(ATCC: 14028), *E. coli *(ATCC: 8739), *S. dysenteriae*(PTCC: 1188),* S. epidermidis*(ATCC:12228), *B. subtilis* (ATCC:6033), MRSA (ATCC:33521) and *P. aeruginosa*(ATCC:9027) were purchased from the Persian Type Culture Collection (PTCC), Tehran, Iran and cultured in SCDA (Soybean-Casein Digest Agar, Merck, Germany) medium and incubated at 37 °C for 24 h.

*Determination of inhibition zone*

Pre-evaluation of antimicrobial effects of the extracts were performed using Cup-Plate method. The microbial suspensions of each bacterium with turbidity correspond to 0.5 McFarland (1×10^8^ Microorganisms) were prepared by normal saline, spectrophotometericaly and spread thoroughly on the surface of plates filled with MHA (Muller-Hinton Agar, Merck, Germany) medium. The wells with diameters equal to 8 mm filled with 100 µL of different concentration of the extracts including: 200, 100, 50, 25 and 12.5 mg/mL dissolved in water/Tween 20 (80:20). Solvent (water and Tween 20 (80:20)) was used as a negative control. The plates were incubated for 24 h at 37 °C. Cup-plate method was performed 2 times and the average diameters of inhibition zones for different concentrations were determined.

*Determination of minimum inhibitory concentration*

Determination of minimum inhibitory concentration (MIC) was utilized by agar dilution method based on National Committee for Clinical Laboratory standards (NCCLS) (40). Extract of each plant was mixed completely with melted MHA to make homogeneous concentrations equal to: 200, 100, 50, 25, 25, 12.5, 6.25, 3.12, 1.56 and 0.78 mg/mL. The bacterial suspensions correspond to 0.5 McFarland were made and diluted 10 times with sterile normal saline. 2 µL of each bacterial suspension was spotted on the surface of each medium. A plate without plant extract was considered as negative control. The plates were incubated at 37 °C for 24 h. MIC evaluation was repeated 2 times more.

*Total phenolics assay*

The amounts of phenolic compounds in the extracts were determined by Folin-Ciocalteu colorimetric method (41). The estimation of phenolic compounds in the extracts was carried out in triplicate and calculated by a calibration curve obtained with Gallic acid (Merck, Germany) as a standard. Total phenolics were expressed in percent as Gallic acid equivalents in dry extract matter.

*Statistical analysis*

Raw data of antibacterial effect was analyzed and compared by GraphPad Prism 5 software using two-way ANOVA and Bonfer ronipost tests. The amounts of total phenolics were compared in five plants using one-way ANOVA and Tukey post hoc.

## Results

In this study, the antimicrobial effects of hydro alcoholic extracts of plants *T. vulgaris*, *T. caramanicus*, *Z. multiflora*, *Z. clinopodioides* and *Z. tenuior* against four foodborne bacteria: *S. aureus*, *S. typhimurium*, *E. coli *and *S. dysenteriae *and four more bacteria including:* S. epidermidis*, *B. subtilis*, MRSA and *P. aeruginosa *were investigated. Pre-evaluation of antimicrobial effect of the extracts using cup-plate technique showed that in all extracts at minimum concentration of 50 mg/mL and even more, inhibition zone diameters were considerable (Table 1). The diameters of inhibition zones of the extracts dilutions were increased with respect to the concentration of the extracts.

**Table 1:** The mean (±SD) of inhibition zone diameter of extracts in different bacteria (mm), n=2.

Plant	**Concentration of extract (mg/mL)**	**S. aureus**	**E. coli**	**S. typhimurium**	**S. dysenteriae**	**B. subtilis**	**S. epidermidis**	**MRSA**	**P. aeruginosa**
T. vulgaris	12.5	11.5 ± 0.5	18 ± 0	17 ±0	12.5 ± 0.5	8 ± 0	17.5 ± 0.5	20 ± 0	11.5 ± 0.5
	25	12 ± 1	18 ± 0	21.5 ± 1.5	14 ± 0	8 ± 0	22.5 ± 0.5	25.5 ± 0.5	12 ± 1
	50	12.5 ± 0.5	20.5 ± 0.5	23.5 ± 0.5	16.5 ± 0.5	11.5 ± 0.5	25 ± 0	22 ± 0	14.5 ± 0.5
	100	13 ± 0	22.5 ± 0.5	25.5 ± 0.5	16.5 ± 0.5	13.5 ± 1.5	35 ± 0	24.5 ±0.5	21.5 ± 1.5
	200	15.5 ± 0.5	26.5 ± 1.5	27.5 ± 0.5	18 ± 0	18 ± 0	38 ± 0	28.5 ± 0.5	29 ± 1
T. caramanicus	12.5	17.5 ± 0.5	8 ± 0	26 ± 4	14 ± 1	13.5 ± 0.5	32 ± 0	20.5 ± 1.5	16 ± 1
	25	16.5 ± 0.5	8 ± 0	21.5 ± 0.5	17.5 ± 0.5	15 ± 0	36 ± 0	21 ± 1	17 ± 1
	50	19 ± 1	14.5 ± 1.5	24 ± 0	18 ± 0.5	16 ± 0	40 ± 0	27 ± 1	23.5 ± 0.5
	100	22.5 ± 0.5	20 ± 0	25.5 ± 0.5	17 ± 0	18 ± 0	40 ± 0	33 ± 1	33 ± 3
	200	24 ± 1	28 ± 2	27.5 ± 0.5	18.5 ± 1.5	2 ± 0	40 ± 0	35.5 ± 0.5	39 ± 1
Z. multiflora	12.5	18 ± 0	10 ± 1	18 ± 0	17 ± 0	17 ± 0	30 ± 0	1.4 ± 1	13 ± 0
	25	20.5 ± 0.5	12 ± 1	20 ± 0	19.5 ± 0.5	19 ± 1	30.5 ± 0.5	1.5 ± 0	16 ± 1
	50	20 ± 1	13.5 ± 0.5	21 ± 0	20 ± 0	16 ± 0	35 ± 1	21 ± 1	20 ± 0
	100	25.5 ± 0.5	16 ± 1	24 ± 0	21 ± 1	2 ± 0	40 ± 0	25.5 ± 0.5	35 ± 0
	200	25.5 ± 0.5	18 ± 2	27.5 ± 0.5	25 ± 1	23 ± 1	40 ± 0	26 ± 1	40 ± 0
Z. clinopodioides	12.5	11 ± 0	8 ± 0	13.5 ± 0.5	13 ± 0	8 ± 0	19 ± 1	13 ± 0	14 ± 1
	25	13 ± 0	8 ± 0	15.5 ± 0.5	15 ± 0	9 ± 1	24.5 ± 0.5	15 ± 1	15.5 ± 0.5
	50	14 ± 0	9.5 ± 0.5	16 ± 0	15 ± 0	12 ± 1	25.5 ± 0.5	19.5 ± 0.5	16 ± 0
	100	17 ± 0	11 ± 1	18 ± 0	16 ± 0	13 ± 1	27 ± 0	22 ± 2	18 ± 0
	200	20.5 ± 0.5	12 ± 1	22 ± 0	18 ± 0	14 ± 1	3 ± 0	25 ± 1	19 ± 1
Z. tenuior	12.5	8 ± 0	8 ± 0	8 ± 0	10 ± 0	8 ± 0	8 ± 0	8 ± 0	14.5 ± 0.5
	25	8 ± 0	8 ± 0	10 ± 0	13.5 ± 0.5	8 ± 0	13 ± 0	8 ± 0	18 ± 0
	50	11 ± 1	9.5 ± 0.5	13 ± 1	14 ± 1	14 ± 1	15 ± 0	11 ± 0	18 ± 0
	100	12.5 ± 0.5	10 ± 1	15 ± 1	15 ± 0	16.5 ± 0.5	19.5 ± 0.5	12.5 ± 0.5	19 ± 0
	200	14.5 ± 0.5	11.5 ± 0.5	16 ± 1	18 ± 1	18 ± 1	21.5 ± 0.5	16 ± 0	2 ± 0

In order to determine the MIC values, two fold serial dilutions from concentration of 200 mg/mL to 0.78 mg/mL were used. The MIC values for *T. caramanicus *and *Z. multiflora *against *S. aureus *were equal to 0.78 and 1.30 mg/mL respectively and less than MIC values of *Z. clinopodioides* and *Z. tenuior*. Antibacterial effect of *Z. multiflora*, *T. caramanicus* and *T. vulgaris *against *E. coli *were more than two other plants with MIC values equal to 1.56, 1.30 and 6.25 mg/mL respectively. *T. caramanicus* and *Z. multiflora *had the most antibacterial effect against *S. dysenteriae, B. subtilis *and MRSA with MIC values ranging between 0.78-3.125 mg/mL.* Z. clinopodioides *and *Z. tenuior* had equal and least antibacterial effect against *S. aureus*, *E. coli*, *S. dysenteriae*,* S. epidermidis*, *B. subtilis*, MRSA and *P. aeruginosa*. Effect of *T. vulgaris *extract against *S. aureus*, *S. dysenteriae*,* S. epidermidis*, *B. subtilis*, MRSA and *P. aeruginosa* was similar to *Z. tenuior *and *Z. clinopodioides *and more than others. Further details are given in Table 2 and Table 3. The rate of antibacterial effect of the plants were compared according to their MIC values using two-way ANOVA and Bonferroni post tests and the p-value less than 0.05 were assumed as significant difference in antimicrobial effect. The results are shown in Table 3.

**Table 2:** The mean of minimum inhibitory concentration (MIC) of extracts in different bacteria (mg/mL), n=3.

**Bac.**	**T. vulgaris**	**T. caramanicus**	**Z. multiflora**	**Z. clinopodioides**	**Z. tenuior**	**Control**
S. aureus	6.25	0.78	1.30	12.50	12.50	8
E. coli	6.25	1.56	1.30	16.66	20.83	8
S. typhimurium	12.50	2.60	1.56	50.00	25.00	8
S. dysenteriae	12.50	0.78	1.56	12.50	10.41	8
B. subtilis	10.41	1.56	1.30	16.66	12.50	8
S. epidermidis	6.25	1.04	1.56	12.50	10.41	8
MRSA	12.50	2.08	3.125	12.50	16.66	8
P. aeruginosa	6.25	1.30	1.56	12.50	10.41	8

**Table 3:** comparison of the mean of minimum inhibitory concentration (MIC) of extracts in different bacteria (mg/mL).

**PLants**	**S. aureus**	**E. coli**	**S. typhimurium**	**S. dysenteriae**	**B. subtilis**	**S. epidermidis**	**MRSA**	**P. aeruginosa**
T. vulgaris	NS	NS				NS		NS
T. caramanicus								
T. vulgaris	NS	NS				NS		NS
Z. multiflora								
T. vulgaris	NS			NS	NS	NS	NS	NS
Z. clinopodioides								
T. vulgaris	NS			NS	NS	NS	NS	NS
Z. tenuior								
T. caramanicus	NS	NS	NS	NS	NS	NS	NS	NS
Z. multiflora								
T. caramanicus								
Z. clinopodioides								
T. caramanicus								
Z. tenuior								
Z. multiflora								
Z. tenuior								
Z. multiflora								
Z. clinopodioides								
Z. clinopodioides	NS	NS		NS	NS	NS	NS	NS
Z. tenuior								

NS: p-value > 0.05

****: p-value < 0.0001

***: p-value < 0.001

**: ‌ p-value < 0.01

**Figure 1:**
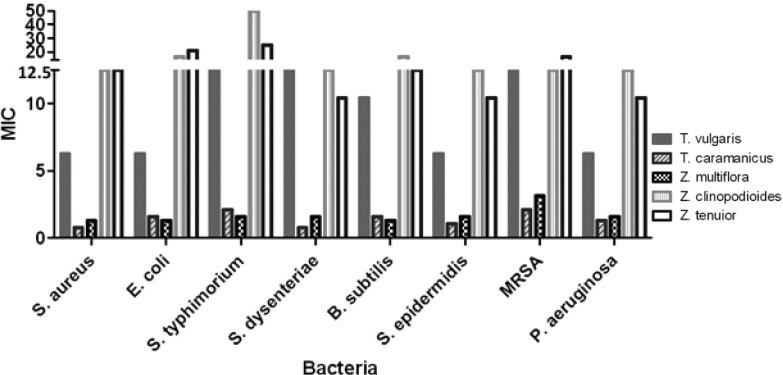
The minimum inhibitory concentration (MIC) of extracts in defferent bacterial species

The amount of total phenolics were evaluated by Folin-Ciocalteu colorimetric method and ranged from 3.785 to 10.247% of dry extract matter. The highest levels were detected in *T. caramanicus* and *Z. multiflora* with non-significant different values equal to 10.247 and 10.192 respectively (P>0.05). The phenolic content in other three species (*T. vulgaris*, *Z. tenuior* and *Z. clinopodioides*) was significantly different with values equal to 8.337, 4.007 and 3.785% respectively (P<0.001). p-value less than 0.05 were assumed as significant different in phenolic content (Figure 1).

## Discussion

Increasing resistance to antimicrobial agents and finding new and relatively low-risk compounds from different natural plants, oriented researchers to evaluate the effect of plants and their active compounds. On the other hand comparing the antibacterial effect of these plants is important for choosing the most appropriate ones. In this study, the effect of hydroalcoholic extract of five species of Labiatae family against four foodborne and some other bacteria were investigated and compared.

The plants of genera *Thymus*, *Zataria and Ziziphora* contain many phytochemical substances including terpenoids and phenolics (32,36,42-44). Chemical compositions of these genera are fairly known at least about two of the most important secondary compounds; volatile oils and phenolic compounds. On the other hand, these two classes of compounds are responsible for the most pharmacological effects of these three genera (39,45,46). Among terpenoids, the phenolic terpenes; thymol and carvacrol, rank highest in importance (21). Studies have shown that these phenolic compounds, especially carvacrol (a main constituent of Avishan-e-Shirazi and Avishan-e-Kermani essential oils) have a high antimicrobial effect (35,47).

According to the results obtained in this study, the antimicrobial effects of the extracts and observed differences may be due to other compounds such as phenolics. On the other hand, total phenolic content determined by Folin-Ciocalteu method is not an absolute measurement of the amount of phenolic materials (48) and it is possible that low-concentration components or interaction between some of the constituents are responsible for the antimicrobial effects (49). It is well-known that phenolic compounds contribute to quality and nutritional value in terms of modifying color, taste, aroma, and flavor and also in providing health-beneficial effects. They also serve in plant defense mechanisms to counteract reactive oxygen species (ROS) in order to survive and prevent molecular damage and damage by microorganisms, insects, and herbivores (48-52).

In general, the differences among the effects of these five plants are probably related to the amount and type of the phenolic compounds, remaining volatile compounds, trace compounds or maybe interaction between constituents.

Regardless of phytochemical studies and molecular composition of these plants, due to the common application of this plant as food condiments, the antimicrobial properties of these plants as a raw and crude material could be discussed. However further studies in order to evaluate the direct effect of these plants extracts or their intact and crude samples on the stability of foods and beverages are needed.

Among evaluated plants, at least two including *T. caramanicus* (Avishan-e-Kermani) and *Z. multiflora* (Avishan-e-Shirazi) have a remarkable antimicrobial effect against foodborne and other bacteria. Study of direct effect of powdered crude plant samples or their extracts on the stability of food is suggested.
